# Tau and Caspase 3 as Targets for Neuroprotection

**DOI:** 10.1155/2012/493670

**Published:** 2012-05-30

**Authors:** Anat Idan-Feldman, Regina Ostritsky, Illana Gozes

**Affiliations:** The Adams Super Center for Brain Studies, The Lily and Avraham Gildor Chair for The Investigation of Growth Factors, The Elton Laboratory for Molecular Neuroendocrinology, and Department of Human Molecular Genetics and Biochemistry, Sagol School of Neuroscience, Sackler Faculty of Medicine, Tel Aviv University, 69978 Tel Aviv, Israel

## Abstract

The peptide drug candidate NAP (davunetide) has demonstrated protective effects in various *in vivo* and *in vitro* models of neurodegeneration. NAP was shown to reduce tau hyperphosphorylation as well as to prevent caspase-3 activation and cytochrome-3 release from mitochondria, both characteristic of apoptotic cell death. Recent studies suggest that caspases may play a role in tau pathology. The purpose of this study was to evaluate the effect of NAP on tau hyperphosphorylation and caspase activity in the same biological system. Our experimental setup used primary neuronal cultures subjected to oxygen-glucose deprivation (OGD), with and without NAP or caspase inhibitor. Cell viability was assessed by measuring mitochondrial activity (MTS assay), and immunoblots were used for analyzing protein level. It was shown that apoptosis was responsible for all cell death occurring following ischemia, and NAP treatment showed a concentration-dependent protection from cell death. Ischemia caused an increase in the levels of active caspase-3 and hyperphosphorylated tau, both of which were prevented by either NAP or caspase-inhibitor treatment. Our data suggest that, in this model system, caspase activation may be an upstream event to tau hyperphosphorylation, although additional studies will be required to fully elucidate the cascade of events.

## 1. Introduction

NAP is an 8 amino acid peptide, which was identified as an active neuroprotective fragment of activity-dependent neuroprotective protein (ADNP) [1]. NAP was found to be neuroprotective in various in vivo and in vitro models of neurodegeneration [2]. NAP treatment was shown to reduce two major pathological markers—tau hyperphosphorylation [3–5] and caspase-3 activation/apoptosis [6, 7]. Hyperphosphorylated and aggregated tau, originally detected in Alzheimer's disease (AD) brains by Grundke-Iqbal and colleagues [8], is a hallmark of a group of diseases, generally referred to as “tauopathies” which differ from each other by genetic background and by additional pathological and phenotypic characteristics [9].

Tau is a microtubule-associated protein (MAP) which promotes microtubule stabilization. The first study that reported disassembly of microtubules from AD brain due to the abnormal hyperphosphorylation of tau was by Iqbal et al. [10]. Hyperphosphorylated tau loses its microtubule affinity causing a change in microtubule dynamics towards disassembly [11–16] and further accumulation of aggregated tau. Alonso et al. originally showed that AD abnormal hyperphosphorylation of tau causes not only loss of function but also the gain of toxic function, with hyperphosphorylated tau blocking microtubule assembly in the presence of normal tau [17] and promoting the formation of normal tau containing tangles [18]. The most prevalent tauopathy is Alzheimer's disease (AD), and other tauopathies include a selection of frontotemporal dementia/degeneration, with pure tauopathies like progressive supranuclear palsy (PSP) [19].

One of the hallmarks of tauopathies is the accumulation of neurofibrillary tangles (NFTs). NFT accumulation correlates with the severity of dementia and memory loss [20–22] and with neuronal degeneration in AD and PSP [23, 24]. Recent studies associated the spread of tauopathies with propagation of prion-like protein inclusions [25] and suggested transsynaptic spread of tau [26]. However, several studies showed that soluble defective tau is also correlated with cognitive deficits [27–29], causing synaptic loss and gliosis before NFTs formation [30]. Tau is also a substrate of multiple caspases, which cleave it and promote its pathologic aggregation [31–33]. Cleaved and hyperphosphorylated tau is found in deposits in AD brains, and it was found that truncated tau promotes apoptosis [34]. The relationship between tau hyperphosphorylation and cleavage is not completely understood. Though some evidence suggests that caspase cleavage of tau is not necessary for tau hyperphosphorylation, this question should be further clarified [35, 36]. Caspases are known to play a role in AD (for review, see [37]), and caspase effects on modifications of tau are of a great interest. Furthermore, treatment of an AD mouse model with a broad-spectrum caspase inhibitor was lately shown to reduce tau pathology but not amyloid-*β* (A*β*) pathology [38].

To date, we have tested NAP effects on either tau hyperphosphorylation or on caspase activation in separate models, showing inhibition of tau pathology or inhibition of caspase activation (or other apoptotic markers) (Table 1). The data emerging from these studies raise the question whether there is a dependency between the effects of NAP on the two pathologies. In the current study, we used an in vitro model of ischemia in which NAP treatment reduced apoptosis [39]. As animal models of ischemia exhibit tau hyperphosphorylation [40–42], we investigated whether the in vitro ischemia model mimics the in vivo situation. Thus, the aim of the current study was to identify a possible interaction between caspase activity and tau hyperphosphorylation, and to test the effect of NAP on both events under ischemic conditions.

## 2. Materials and Methods

All procedures involving animals were approved by the Animal Care Committee of Tel-Aviv University and further approved by the Israel Health Authorities.

### 2.1. Primary Neuronal Cultures

Primary neuronal cultures were produced from cortices obtained from 1-2-day-old Sprague-Dawley rat pups. Pups were decapitated, their skulls were removed, and their cortices were transferred to a Petri dish field with HBSS-HEPES buffer. Meninges were removed using tweezers under binocular. Neurons were produced with Worthington's papain dissociation kit (Worthington, cat.LK003150) according to the manufacturer's instructions. The cells were seeded in Neurobasal-A media (Invitrogen, cat. 10888022) fortified with 1% Glutamax supplement (Invitrogen, cat. 35050038) and 1% Neurocalm (Stemcells technologies, cat. 05711). All tests were executed 5-6 days in vitro (DIV).

### 2.2. Antibodies

The following antibodies were used for immunefluorescence: NeuN (Neuronal Nuclei, Chemicon, cat. MAB377), diluted 1 : 100, and GFAP (glial fibrillary acidic protein, Abcam, cat. 7260), diluted 1 : 500. The following antibodies were used for immunoblot analysis: p-tau^202^ (Anaspec, cat. 28017) 0.25 *μ*g/mL, total tau (MBL international, cat. AT-5004) 0.5 *μ*g/mL, and active caspase-3 (Abcam, cat. 2302) 1 *μ*g/mL. Fluorophore and horseradish peroxidase conjugated secondary antibodies were purchased from Jackson ImmunoResearch Laboratories Inc.

### 2.3. Analysis of Culture Purity

Cells were seeded in 24 well plates on poly-D lysine- (PDL-) coated coverslips (Sigma-Aldrich, cat. p-6407) at a density of 0.5∗10^5^ cells/well. At 5–6 DIV, the cultures were fixed using a 4% paraformaldehyde (PFA) solution. Antigen retrieval, if needed, was performed by boiling the sample in sodium citrate buffer (pH = 6) for 20 minutes. Permeabilization was performed with a 0.2% Triton-X solution. Nonspecific antigen binding was blocked by incubation in a 2% bovine serum albumin (BSA) solution, then primary antibody (Ab) diluted in primary antibody diluent (Biotech applications, cat. MSBA-AbDil) was added for 1 hour at RT. Cells were washed three times in 2% solution of BSA and incubated in secondary antibody solution for 30 minutes in the dark. After an additional wash, PBS + DAPI (4′,6-diamidino-2-phenylindole, a fluorescent stain that binds strongly to A–T-rich regions in DNA, Invitrogen) was applied for 5 minutes, in order to stain cell nuclei. The coverslips were put on an antifading buffer on a carrier slides. For analysis of culture purity, we used 3 cultures produced independently. We used Zeiss fluorescence microscope and camera to obtain 10 pictures (×20 magnification) from each culture. Each picture contained a minimum of 30 cells. NeuN-positive and GFAP-negative cells were considered neuronal and were expressed as % of the total DAPI count.

### 2.4. OGD Treatment

Cells were seeded on PDL-coated plates employing two different plating conditions (Corning): (1) 10 cm Petri dishes, 6∗10^6^ cells per dish (for protein extraction), and (2) 48 well plates 0.5∗10^5^ cells/well (for viability tests). After 5-6 DIV, the condition media (CM) was collected, filtered, and kept at 37°C. Cultures were washed twice with 37°C PBS, and the experimental media was added (PBS for the OGD groups, fresh media for the control group). Treatments were added to the experimental media (QVD-OPH, a broad-spectrum caspase inhibitor [43], 20 *μ*M diluted in DMSO (Biovision, cat. no. 1170) or 10^−5^ M NAP diluted in PBS or diluents only). The culture dishes were placed in an ischemic chamber (Billups-Rothenberg, Inc., cat. MIC 101) which was filled with a 5%CO_2_/95%N_2_ gas mixture and sealed, and the chamber was placed in an incubator for 2 hours; at 37°C. Two experimental modes were applied, in the first one (used for NAP protecting concentration curve), gassing was for 25 minutes followed by sealing for 2 hours, in the second paradigm (used for calibration of caspase inhibition and protein analysis), gassing was for 5 minutes only. O_2_ percentage was monitored with O_2_ meter (HUMIL International Corporation, cat. PO2-250) and did not exceed 1.2% any time during the OGD period. At the end of the OGD period, the culture dishes were removed from the chamber. For protein extraction, the experimental media was removed and protein was extracted as described below. 

### 2.5. Viability Assay

Cells were seeded in 48 well plates at a density of 0.5∗10^5^ cells/well (as described above). Following the OGD period, the condition media (CM) which was removed at the initiation of the OGD treatment (see above) was reapplied to the corresponding treatments. The MTS assay was used to assess viability (Promega, cat. G3580). Absorbance at 490 nm was measured in a SpectraMAX 190 plate reader, (Molecular Devices, Inc.). The results were analyzed using SoftMax Pro software version/year (Molecular Devices, Inc.) and expressed as % of the control. The results summarize 3 independent experiments with 5 replicates per treatment per experiment. Data are presented as means/medians ± SE.

### 2.6. Immunoblots

Immediately after OGD treatment (following gassing mode of 5 minutes of gassing and 2 hours OGD period), experimental media was discarded, and the dishes were washed twice with PBS. Protein was extracted using RIPA buffer with anti-proteases (ROCHE, cat. 11 873 580 001) and anti-phosphatases. Protein concentration was quantified using Pierce BCA protein assay reagents (Pierce, cat. 23223, 23224) with bovine serum albumin (BSA) as a standard protein. Equal amounts of protein diluted in sample buffer were separated by sodium dodecyl sulfate (SDS) polyacrylamide gel electrophoresis (12% gels or 4–20% gradient gels, nUView, cat. NG21-420). Proteins were transferred to nitrocellulose membranes using standard techniques, as before [44]. Membranes were blocked with 5% BSA in Tris-buffered saline containing 0.1% Tween-20 (TBSt) and then incubated overnight with primary antibody. Membranes were washed 3 times with TBSt and incubated with secondary Ab for 1 hour at RT. Pierce ELC substrate or Pierce super signal ECL was used to visualize proteins (cat. 32106, 34080). Signal density was detected with the DNR bioimaging systemsMiniBIS pro (DNR Bio-Imaging Systems Ltd.) and TotalLab TL-100 software (Nonlinear Dynamics Ltd.).

### 2.7. Statistical Analysis

SPSS 19.0 for windows (http://www-01.ibm.com/software/analytics/spss/) was used for statistical analysis. For viability assessment, 3 independent experiments were conducted with 5 trials per treatment in each experiment. Data were analyzed using a one-way ANOVA with 5% selected the level of significance, and post hoc Scheffe and LSD corrections. The effect of NAP was evaluated in 14 independent experiments, of which in 4 experiments QVD-OPH effect was also tested, and the levels of significance were evaluated using ANOVA with post hoc LSD.

## 3. Results

### 3.1. Characterization of Culture Purity

**Figure 1:**
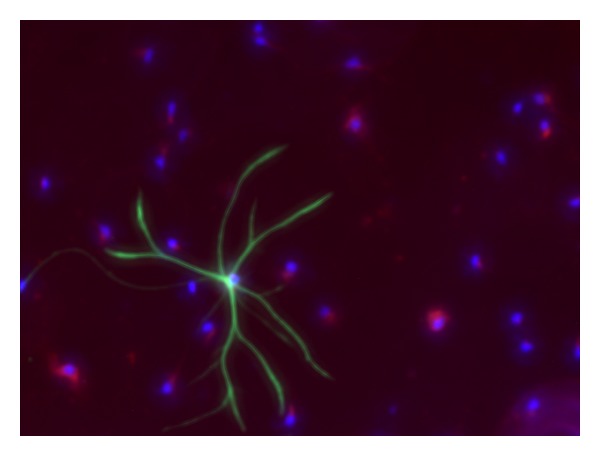
Culture purity: at 5-6 DIV, cells were fixed and immunofluorescence staining was performed using the astrocyte marker GFAP (green), the neuronal marker NeuN (red), and DAPI stain (blue) for nuclei. Quantification of neuronal cells was done using 10 random fields from each of 3 experiments (×20 magnification). 95.9 ± 0.96% of the total cells (DAPI stain) were recognized as neurons (NeuN positive, GFAP negative).

Culture purity was analyzed using immunofluorescent staining with cell-specific markers. After 5-6 DIV (days in vitro), 95.9 ± 0.96% (SEM) of the cells (counted using nuclear DAPI stain) were positive for the neuronal NeuN marker and negative for the astrocytic marker, GFAP. A representative immunefluorescence stain in Figure 1 shows neuronal cells stained with NeuN in red, and astrocytes stained with GFAP in green. 

### 3.2. NAP Protective Effect following 2 Hours of OGD

**Figure 2:**
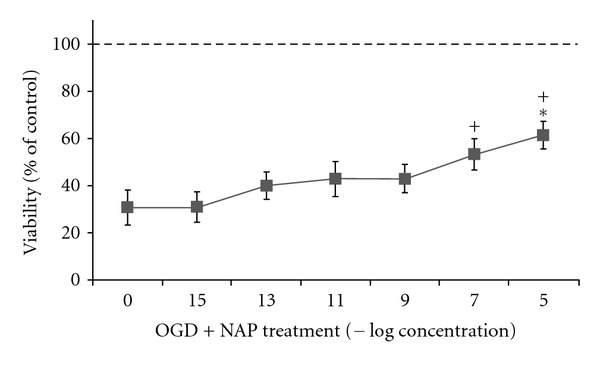
NAP protects from cell death in a dose dependent manner following 2 hours of OGD insult, but not after additional 24 hours reperfusion. Primary neuronal cultures (5-6DIV) were subjected to 2 h of ischemic insult. Cell viability was evaluated using MTS viability assay. Data was normalized to % of control. Results are shown as mean ± SE. *Significantly different from OGD with no NAP, OGD + NAP10^−15^ M, OGD + NAP 10^−13^ M, OGD+NAP 10^−11^ M (3 independent experiments are summarized, ANOVA, with post hoc Scheffe, significant difference considered to be *P* ≤ 0.05). Using LSD post hoc, NAP 10^−5^ M (+), 10^−7^ M are significantly different from the OGD group (*P* < 0.05).

Cell viability was evaluated following a 25-minute gassing period and an additional 2 hours of OGD. The results were calculated and expressed as % of the control group (Figure 2). Under this paradigm, ~70% cell death was observed. Treatment with NAP increased cell viability immediately following OGD (*F* = 17.667, df = 7, *P* < 0.001). Post hoc pairwise comparisons (LSD) indicated that NAP concentrations of 10^−7^ M and 10^−5^ M significantly protected against ischemia (Figure 2, *P* ≤ 0.05). 

### 3.3. Apoptosis Was the Main Type of Cell-Death Following 2 Hours of OGD

**Figure 3:**
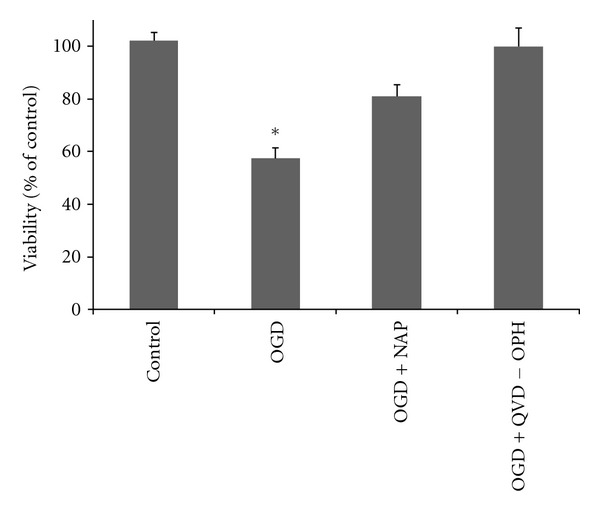
Following 2 hours of OGD, cells died exclusively from apoptosis. Cultures were treated with either NAP (10^−5^ M) or QVD-OPH (2∗10^−5^ M) and subjected to 2 h of ischemic insult. Cell viability was evaluated immediately following the ischemic period using MTS viability assay. Data was normalized to % of control. Results are shown as mean ± SE; *significantly different from all other groups, *P* ≤ 0.05 (ANOVA, with post hoc Scheffe).

Using the broad spectrum caspase inhibitor QVD-OPH, we tested the extent of apoptotic death following 5-minute of gassing and 2 hours of OGD. Cell viability was evaluated and compared to the control group (Figure 3). Under this paradigm, ~55% cell death was observed. Here, QVD-OPH (2∗10^−5^ M) treatment significantly increased cell viability to the control levels. In the same experiment, NAP in the same experiment, NAP (10^−5^ M) treatment rescued from the apoptotic cell death (*F* = 16.090, df = 7, *P* < 0.001). Post hoc pairwise comparisons did not detect any significant differences between the caspase inhibitor, NAP, and control no-ischemia groups.

### 3.4. Active Caspase-3 Expression Was Increased Following OGD and Reduced Due to NAP Treatment

**Figure 4:**
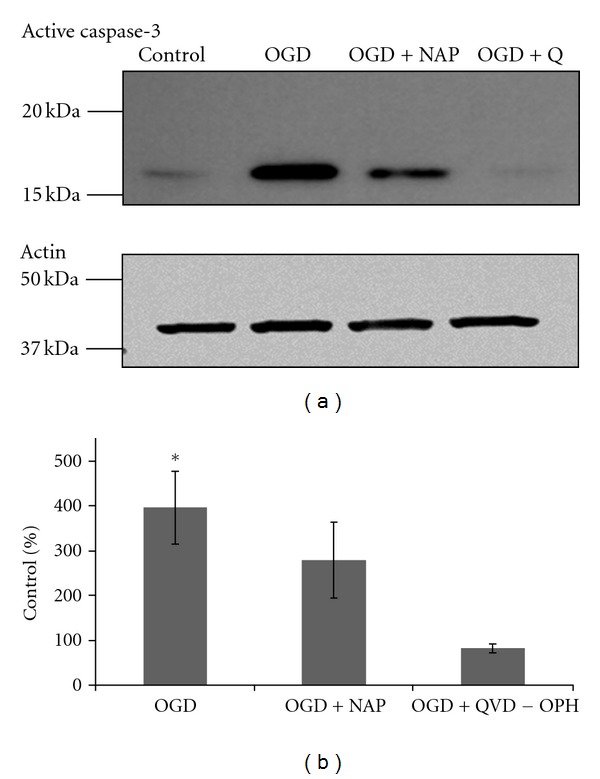
An increase in active caspase-3 levels induced by 2 hours of OGD was diminished by NAP treatment (10^−5^ M). Cultures were treated with 10^−5^  M NAP or with 20 *μ*M QVD-OPH (broad spectrum caspase inhibitor) exposed to OGD insult for 2 hours. Proteins were extracted and analyzed using immunoblot with a specific antiactive caspase-3 antibody. A representative blot of active caspase-3 antibody is exhibited in (a). (ANOVA with post hoc LSD, *P* ≤ 0.05) (b).

The levels of active caspase-3 were evaluated immediately after the OGD period using immunoblots with a specific active caspase-3 antibody and further quantified and normalized to actin. As shown in a representative blot in Figure 4(a), OGD treatment increased active caspases-3 levels. This increase was prevented by NAP treatment (10^−5^ M) or QVD-OPH (caspase inhibitor) treatment (2∗10^−5^ M).  Figure 4(b) depicts the level of active caspase-3 expressed as % of control. A significant increase in active caspase-3 expression was induced by OGD and partially but significantly prevented by NAP treatment (*F* = 7.880, df = 2,  *P* = 0.004).

### 3.5. Tau Hyperphosphorylation Was Increased Following 2 Hours of OGD; P-Tau Increase Was Prevented by Either NAP Treatment or QVD-OPH Treatment

**Figure 5:**
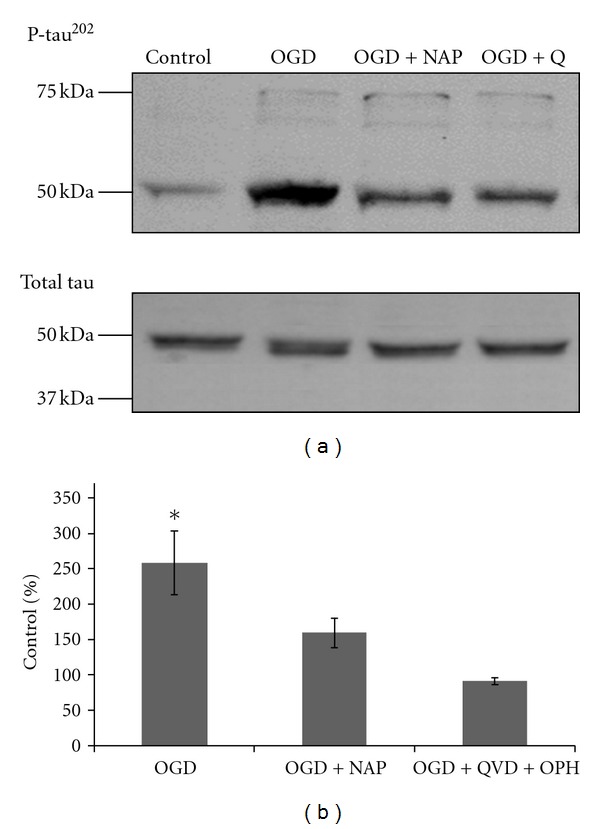
2 hours of OGD caused an increase in p-tau^202^ levels, prevented by either NAP or QVD-OPH treatment. Cultures were treated with 10^−5^ M NAP or with 20 *μ*M QVD-OPH (broad spectrum caspase inhibitor, indicated as OGD + Q) and exposed to OGD insult for 2 hours. Proteins were extracted and analyzed using immunoblot with a specific anti-p-tau^202^ and antitotal tau antibodies. A representative blot of p-tau^202^ and total tau is exhibited in (a). P-tau levels were quantified and normalized to total tau levels (ANOVA with post hoc LSD, *P* ≤ 0.05) (b).

phospho-tau (p-tau) levels were detected by immunoblot, with a p-tau^202^-specific antibody. A representative blot is shown in Figure 5(a). P-tau levels, were quantified, normalized to total tau levels and expressed as % of the p-tau levels in the control culture (Figure 5(b)). Phospho-tau levels were significantly increased following the OGD insult. Both NAP treatment (10^−5^ M) and QVD-OPH (caspases inhibitor) treatment (2∗10^−5^ M) prevented p-tau increase (ANOVA with post hoc LSD, *P* ≤ 0.05), (*F* = 5.633, df = 2, *P* = 0.012).

## 4. Discussion

Focusing on the protective properties of NAP, two central characteristics of neurodegeneration have been shown to be inhibited by NAP treatment both in vivo and in vitro: tau hyperphosphorylation [3–5] and apoptosis [6, 7, 51]. The main goal of the current study was to look for an association between these two pathological cascades. Our experimental setup used primary neuronal cultures (95.9 ± 0.96% purity), subjected to 2 hours of OGD with no reperfusion period. Under this paradigm, apoptosis was found to be responsible for all cell death in that it was inhibited by QVD-OPH. Our results showed that inhibition of caspase activity prevented tau hyperphosphorylation, leading us to conclude that in the current experimental and cellular conditions, caspase activation is an upstream event to tau hyperphosphorylation. We further evaluated the effect of NAP on cell viability, caspase-3 activation, and tau hyperphosphorylation. Viability assays showed that NAP treatment rescued cells from apoptosis as demonstrated by the reduction in active caspases-3 following NAP treatment. NAP treatment also prevented tau hyperphosphorylation after the OGD. Combining the effect of NAP on active caspases-3 and tau hyperphosphorylation, it seems likely that caspases-3 or an upstream pathway is targeted by NAP activity in isolated neuronal cells that are metabolically stressed. 

A careful look should be given to the concentrations by which NAP had a significant protective effect. Previous data, including ischemia-reperfusion experiments conducted in primary neuronal and neuroglial cultures, exhibited protection from cytochrome-c release, microtubule breakdown and reduction in MAP2 intensity using NAP concentrations of 10^−15^ M–10^−8^ M [39, 52]. Though most studies used femtomolar concentration of NAP, Pascual and Guerri [53] used concentration of 10^−7 ^M to show NAP protection in a model neurons cocultured with astrocytes obtained from prenatal ethanol-exposed fetuses. In our model, protection was detected when treating with NAP at concentrations ≥10^−7^ M. The differences in the potent NAP concentration could be explained by the combination of the acute insult applied to a relatively pure culture of immature neurons. Immature neurons (2–4 DIV) in a pure neuronal culture (99% purity) were shown to go through spontaneous apoptotic death which was prevented when astrocyte condition media was added to the neuronal cultures. In comparison to neuroglial mixed culture, pure neuronal cultures were also found to be more sensitive to excitotoxic insult that also plays a role in the ischemia induced cell death [54]. We have previously shown that neuronal protection in the absence of glia required increased concentrations of NAP as compared to mixed neuroglial cultures [55]. Furthermore, the original discovery of the NAP containing protein, ADNP, was as a glial protein providing neuroprotection. ADNP is essential for central nervous system development, expressed in specific brain tissue in the adult brain [1], and is also deregulated and may be overexpressed following acute brain damage [56] and in animal models of neurodegeneration [57, 58]. ADNP-like immunoreactivity is secreted from glial cells [59] further providing neuronal protection [4, 53, 60]. In this respect, NAP protected against ADNP deficiencies as outlined in the Introduction [4]. NAP was further shown to promote neurite outgrowth [61, 62] provide microtubule stabilization [63] and protect neurons in a broad spectrum of neuropathologies models [2]. Importantly, NAP also showed glial protection in vitro [64] and in vivo [7].

In summary, we demonstrated that NAP reduces caspase-3 activation, and tau hyperphosphorylation, suggesting that caspase-3 or upstream pathways may be targets for NAP activity. We believe these data provide additional insights regarding the molecular mechanism for NAP's neuroprotective activity and the intricate interaction between microtubules/tau and apoptotic mechanisms.

## Figures and Tables

**Table 1:** Models of NAP protection against tau pathology or increased markers of apoptosis.

Models of tau pathology detecting protective effects when treated with NAP
(i) Transgenic ADNP heterozygous mouse [4]
(ii) Transgenic human double-mutant tau mouse [5]
(iii) Triple transgenic mice expressing the amyloid (A*β*) precursor protein APP(Swe), presenilin PS1 (M146V), and tau (P301L) [45, 46]
(iv) Mixed neuroglial primary cultures treated with A*β* (1–42, 2.5 *μ*M) [3]
(v) Primary cultures of astrocytes [47]

Models of apoptosis detecting protective effects when treated with NAP

(i) A stroke model using spontaneously hypertensive rats which underwent permanent middle cerebral artery occlusion [48]
(ii) A rat model of diabetes (streptozocin toxicity) [7]
(iii) A rat model of epilepsy [49]
(iv) PC-12 cells exposed to H_2_O_2_ [50]
(v) Primary neuronal cultures subjected to ischemia/ reperfusion schedule of 3 h/3 h [39]
